# Allostatic Load in Perimenopausal Women With Migraine

**DOI:** 10.3389/fneur.2021.649423

**Published:** 2021-04-22

**Authors:** Pamela Alebna, Nasim Maleki

**Affiliations:** ^1^Department of Epidemiology, Harvard T.H. Chan School of Public Health, Boston, MA, United States; ^2^Department of Psychiatry, Massachusetts General Hospital, Harvard Medical School, Boston, MA, United States

**Keywords:** migraine, menopause, perimenopause, allostatic load, biomarkers, burden, sleep problems

## Abstract

**Objective:** There is very limited data on women with migraine disease as they age and transition to menopause. Despite evidence for the increased burden of the disease during this transition, there is no data on the association between migraine and allostatic load as a marker of cumulative biological risk. We aimed to determine whether women with migraine suffer from higher levels of allostatic load during perimenopausal transition.

**Methods:** A total of 2,105 perimenopausal women from the first wave of the Study of Women's Health Across the Nation (SWAN) were included in this study. Allostatic Load (AL) score was estimated for each participant from the measurements of: systolic and diastolic blood pressure, C-reactive protein level, high-density lipoprotein cholesterol level, total cholesterol level, waist-to-hip ratio, fasting serum glucose, triglycerides, and dehydroepiandrosterone levels.

**Results:** Of the 2,105 participants included in the study, there were 369 migraineurs and 1,730 controls. Migraineurs had 63% higher odds of increased load score (odds ratio 1.63; 95% confidence interval, 1.17–2.29). Compared to controls, migraineurs were more likely to experience sleep problems in the univariate analysis, however despite the high burden of sleep problems, there were no significant associations between allostatic load and sleep disturbances in perimenopausal women with migraine after controlling for other factors.

**Conclusion:** This is the first study to systematically and quantitatively examine allostatic load in migraine patients. The findings establish that migraineurs are more likely to experience higher allostatic load than their non-migraine counterparts during perimenopausal transition. The findings encourage new lines of investigation for lowering the burden of the disease through interventions that modify the levels of allostatic load biomarkers examined in this study.

## Introduction

Migraine is a chronic and debilitating primary headache disorder with global prevalence of 18.9% for women and 9.8% for men (1). In addition to the higher prevalence of migraine among women, they are also more likely to have more chronic migraines and comorbidities, and have more disabling symptoms than men (2). The frequency of migraines is known to change across a woman's lifetime (3) with established linkage to hormonal fluctuations and reproductive milestones (3, 4). Menopausal transition is a particularly prolonged period of strong hormonal fluctuations with the risk of chronic headaches increased by almost 60% in migraineurs (5). In addition, unique features of a woman's menopausal transition may make them more or less prone to increased migraine attacks (4, 6).

Allostatic load (AL), as an assessment of the body's physiological response to stress, both chronic and acute, is commonly described as a measure of the “wear and tear” the human body goes through in the face of internal and external stressors. It seeks to estimate the physiological impact of sustained stress, whether biological or non-biological, on health. The chronic stress model of allostatic load suggests that, when the body is subject to stressors for varying lengths of time, its compensatory mechanisms kick in to maintain homeostasis. If this persists, it results in a dysregulation of biological systems which can be estimated by measuring certain biomarkers (7–9).

In this study we hypothesized that women with migraine experience a significantly higher allostatic profile due to the added burden of frequent headaches and increased sleep disturbances during menopausal transition. This hypothesis is based on the burden of repeated migraine attacks as stressors (10, 11), the central role of the hypothalamically-mediated homeostasis (12) to the pathophysiology of migraine, and the significant modulatory influence of hormonal fluctuations during menopausal transition on the brain (13). Furthermore, since sleep disturbances have long been associated with migraines and other headache disorders (14), and sleep disorders are one of the main symptoms associated with menopausal transition (15–17), we hypothesized that sleep problems will have a modulatory influence on allostatic load and exacerbate it. As research continues to delve into the pathophysiology of migraine, with this study, we hoped to identify potentially modifiable and physiologically quantifiable risk factors that could be targeted to reduce the burden of the migraine disease for women going through menopause.

## Methods

### Study Population

Data for this study was obtained from the Study of Women's Health Across the Nation (SWAN) which is a multi-site prospective study of women's health through menopausal transition consisting of a baseline assessment in 1996–1997 and 10 waves of subsequent annual assessments at all participating sites (18). The institutional review board (IRB) at each of the SWAN study sites had approved the study protocol, and all participants had provided written informed consent prior to participation in the study. The Institutional Review Board (IRB) at the Massachusetts General Hospital also approved the secondary data analysis. Women who participated in the study were eligible to enroll if they: (1) were 42–52 years of age, (2) had an intact uterus and at least one ovary, (3) had at least one menstrual cycle within the past 3 months, and were excluded if they used oral contraceptives or hormone therapy. Analysis was done on data from only wave 1 of the SWAN study which included a total of 2,881 women meeting the eligibility criteria. Of those, 2,105 had all the measurements required for the allostatic load score calculation and were included as the final study sample.

### Migraineurs and Controls

Migraineurs were identified per self-report similar to previous reported studies in this population (12, 19). Participants were asked if a doctor or any other health professional had diagnosed them with migraine headaches. Controls consisted of individuals who reported no history of migraine.

### Allostatic Load Score

Some AL markers have been shown to correlate better with physiological stressors than others. The number of biomarkers selected for inclusion into the estimation varies across studies (20–23), though originally the score was designed with 10 biomarkers (7). In this study a score for allostatic load (AL score) was calculated based on measurements representing cardiovascular (systolic and diastolic blood pressure), inflammatory (C-reactive protein), metabolic (high-density lipoprotein cholesterol, total cholesterol, waist-to-hip ratio, fasting serum glucose, and triglycerides), and neuroendocrine (dehydroepiandrosterone) systems. The AL score was calculated per the cut-off values defined separately for each of the measures based on their distribution so that if the measure was in the upper fourth-quartile of its corresponding distribution it received a high-risk score of 1, and the AL score was calculated by the summation of all the high-risk scores for each individual across all the measures similar to previous studies (24–27).

### Sleep Problems

Three types of reported sleep disturbances were included in the analyses, i.e., trouble falling asleep, waking up several times during the night, and waking up earlier than usual. Each of these problems was considered separately in our analyses.

### Menopausal Status

Menopausal status was determined per the STRAW+10 guidelines (28) based on participants self-reported data on their menstruation patterns and included: pre-menopause, early peri-menopause, late perimenopause, post-menopause, and surgical menopause (through hysterectomy or bilateral salpingo-oophorectomy). In this study, we focused on women with naturally occurring menopausal changes. Therefore, women with surgical menopause were not included in our regression models and analyses.

### Other variables

Demographic variables included age at the time of assessment, race/ethnicity, body mass index (BMI) (categorized as <18.499, underweight; 18.50–24.999, normal; 25–29.999, overweight; and ≥30, obese), and annual household income as a proxy for socioeconomic status (categorized as < $19,999, $20,000–49,999, $50,000–99,999, or ≥ $100,000).

### Statistical Analysis

#### Descriptives

Preliminary analysis involved assessing normality of continuous variables with Shapiro-Wilk test and symmetry using the skewness coefficient. The amount and distribution of missing data between the migraineurs and controls was assessed. The differences in the continuous variables between the two groups was tested with the Independent samples *t*-test or the Wilcoxon rank sum test where appropriate. Chi-square tests were conducted to compare the differences in categorical variables between the migraineurs and controls.

#### Model Building

Key variables that could act as predictors were selected a priori based on subject matter knowledge. In our primary analysis, we first used ordinal logistic regression to fit an unadjusted univariate model of AL score and diagnosis of migraine. Then, two multivariable regression models were constructed for the analyses with: (a) age, race, income, BMI and menopausal status, and with (b) age, income, race, BMI, menopausal status, and form of sleep problem. Sensitivity analysis was also run where AL score was treated as a continuous variable and also where it was categorized from a score with a scale of 9 into a score with a scale of 3 (0≤4 = 1, 5 ≤7 = 2, 8 ≤9 = 3). In the literature, some prior studies have treated AL as continuous variable (20) while others have categorized (23) it into 3 or 4 categories just like what we did in our analyses. For each of the models the proportional odds assumption and the convergence criterion were also assessed. We also tested for effect modification of age, menopausal status and BMI on the association between allostatic load and diagnosis of migraine. Finally, a Poisson regression model was constructed to assess the difference in the sleep problems between migraineurs and controls while adjusting for age, income, BMI, and menopausal status. In all analyses, a two-tailed *P* < 0.05 was considered statistically significant. All statistical analyses were performed with SAS 9.4.

## Results

### Descriptive Characteristics

Of the 2,349 subjects, 244 subjects were excluded due to surgical menopause or missing information on their menopause status. There were 2,105 observations in total, with 369 migraineurs, and 1,730 controls that were included in the final sample. The mean age of the migraineurs and controls were comparable, 46.88 and 46.97 years, respectively. The migraineurs and control subjects were similar in terms of race distribution with the majority of participants being of Caucasian and Black ethnicity in both groups. Caucasian were 58.54% of migraineurs and 46.66% of controls, Blacks were 25.75% of migraineurs and 25.75% of controls, Hispanics 4.88% of migraineurs, and 5.30% of controls and finally Asians were 10.84% and 22.29% of migraineurs and controls, respectively. With regards to menopausal status most of our migraineurs (95.60%) and controls (93.91%) were premenopausal and early premenopausal mainly because we looked at the first cohort of the study at which time most participants had not transitioned to menopause. In general, the baseline characteristics of subjects were similar in both migraineurs and controls (Table 1).

**Table 1:** Baseline Characteristics.

**Characteristics**	**Migraineur *N* = 403**	**Control *N* = 2,025**	***p*-value**
**Age (mean** **±** **SD years)**	46.88 ± 2.81	46.97 ± 2.71	0.592
**Race (*****n*****, %)**			<0.001
Caucasian	216 (58.54%)	810 (46.66%)	
Black	95 (25.75%)	447 (25.75%)	
Hispanic	18 (4.88%)	92 (5.30%)	
Asian (Japanese/Chinese)	40 (10.84%)	387 (22.29%)	
**BMI (*****n*****, %)**			0.097
Normal	127 (34.42%)	685 (39.48%)	
Overweight	109 (29.54%)	459 (26.46%)	
Obese	128 (34.99%)	566 (32.62%)	
Underweight	5 (1.36%)	25 (1.44%)	
**Income (*****n*****, %)**			0.427
<$19,999	45 (12.61%)	171 (10.23%)	
$20,000–$49,999	110 (30.81%)	517 (30.94%)	
$50,000–$99,999	141 (39.50%)	654 (39.14%)	
$100,000 or more	61 (17.09%)	329 (19.69%)	
**Menopausal status (*****n*****, %)**			0.206
Premenopausal	77 (23.91%)	444 (27.91%)	
Early perimenopause	231 (71.74%)	1,050 (66.00%)	
Late perimenopause	11 (3.42%)	72 (4.53%)	
Postmenopausal	3 (0.93%)	25 (1.57%)	
**Sleep problems (mean** **±** **SD)**
Trouble falling asleep	2.4 ± 3.43	1.6 ± 2.75	<0.001
Waking up during sleep	4.1 ± 4.19	3.2 ± 3.87	<0.001
Waking up early	2.5 ± 3.69	1.9 ± 2.92	<0.001

### Missing Data

The distribution of missing data was heterogenous across the variables from 0.01 to 9.1% of the total observations for each variable. Allostatic load was calculated from other biomarkers, hence it had no missing value because missing values in the biomarkers were excluded from the estimation. In general the distribution of missing data between the cases and controls were similar.

### Allostatic Load

We used multivariable models that allowed the assessment of the unique predictive power of selected variables while controlling for other confounding variables in the model. In model 1, after controlling for age, income, BMI, and menopausal status, migraine diagnoses was found to be significantly associated with AL. Comparing migraineurs to controls, there was 63% increased likelihood of a higher allostatic score in migraineurs. Age, race, being overweight, being obese were significant predictors of high AL scores. This model had a predictive probability of 83.1%.

In model 2, age, race, income, BMI, menopausal status, and form of sleep problem were included as predictors. The model had a predictive probability of 83.2%. In this model the odds of a higher AL score was 60% more likely in migraineurs compared to controls. The stage of menopause was not significant in either models, which implies that the effect of migraine on AL was not significantly confounded by the stage of menopause. Table 2 shows the multivariable adjusted OR and 95% confidence intervals for both models. The race and menopause interaction term (*p* = 0.726), BMI and menopause interaction term (*p* = 0.714) as well as the BMI and race interaction term (*p* = 0.147) were not significant, hence there is no evidence of effect modification on the multiplicative scale. However, using allostatic load as a continuous score there was evidence of effect modification between BMI and race (*p* < 0.01). To test if the conclusions from the model would be altered had AL score been treated as a continuous score, as had been done in some studies, another model was run using the 9-score scale as a continuous scale, the results were similar to our original model, this is shown in Appendix 1.

**Table 2:** Ordinal logistic regression models with allostatic load on migraine and other predictors.

	**Model 1**			**Model 2**		
**Effect**	**OR**	**95% Confidence limits**		**OR**	**95% Confidence limits**	
**Migraine**	1.639	1.173	2.292	1.602	1.144	2.244
**Age**	1.052	1.001	1.106	1.052	1.001	1.106
**Race**						
Asian	2.641	1.653	4.220	2.666	1.667	4.264
Black	1.477	1.094	1.994	1.474	1.090	1.994
Hispanic	2.595	1.519	2.870	2.584	1.506	4.432
Caucasian	Ref			Ref		
**BMI**						
Obese	36.711	21.331	63.180	35.869	20.835	61.752
Overweight	7.581	4.391	13.089	7.542	4.369	13.022
Underweight	1.137	0.143	9.052	1.093	0.137	8.737
Normal	Ref					
**Menopause**						
Early perimenopause	1.215	0.886	1.667	1.215	0.884	1.672
Late perimenopause	1.406	0.738	2.680	1.398	0.730	2.675
Post menopause	2.854	1.094	7.766	2.850	1.052	7.726
Premenopausal	Ref					
**Income**						
$20,000–$49,999	0.778	0.514	1.178	0.793	0.524	1.202
$50,000–$99,999	0.757	0.495	1.159	0.785	0.521	1.206
$100,000 or more	0.757	0.317	0.934	0.543	0.315	0.935
<$19,999	Ref					
**Sleep initiation**				1.009	0.971	1.050
**Sleep Maintainance**				1.016	0.964	1.070
**Early Awakening**				1.010	0.959	1.064

*c statistic for model 1: 0.831*.

*c statistic for model 2: 0.832*.

*OR, Odds ratio*.

### Sleep Problems

Compared to controls, migraineurs were more likely to have difficulty falling asleep on most nights (2.4 vs. 1.6 nights, *P* ≤ 0.0001), wake up during sleep (4.1 vs. 3.2 nights, *P* < 0.0001), and to wake up earlier than usual (2.5 vs. 1.9 nights, *P* ≤ 0.0001) in the preceding 14 days, Table 1. After controlling for age, income, BMI, and menopausal status, migraineurs had a higher expected count of nights with sleep problems compared to controls, as reported in Table 3.

**Table 3:** Poisson regression model of the number of nights with reported sleep problems comparing migraineurs to controls.

**Variable**	**Parameter estimate (Days)**	***p*-value**
Difficulty falling asleep	1.95	<0.001
Wake up during sleep	1.34	<0.001
Wake up earlier than usual	1.35	<0.001

*Parameter estimate = number of nights in the preceding 14 days that migraineurs compared to controls had difficulty with sleep after adjusting for race, income, BMI, stage of menopause and age*.

## Discussion

Perimenopausal transition coincides with significant cellular, metabolic, and hormonal changes. These changes could inevitably impair the normal physiological functioning and impact the regulation of homeostasis in the body. The cumulative result of these changes and the consequent adaptation to restore homeostasis could lead to increased allostatic load (29). Individuals with migraine could particularly be more sensitive to homeostatic dysregulation and consequent increase in allostatic load as findings from several clinical and preclinical studies suggest that migraines likely result from dysfunctional homeostatic mechanisms (30). Moreover, the occurrence of migraine attacks in one's life course could further serve as a stressor and contribute to the “wear” and “tear” in the nervous system. In this study of 2,428 perimenopausal women, we found a positive association between migraine and allostatic load which aligns with findings in previously conceptualized models (10, 11). We also found that perimenopausal migraineurs are more likely to have sleep problems such as difficulty falling asleep on most nights, waking up during sleep, and also waking up earlier than usual. However, there was no modification of the association between AL and migraine by sleep.

While measurement of allostatic load through calculating AL score, is one of the most frequently used methods to assess the physiologic response to stress, the number of biomarkers included are not consistent across all studies (20). What is consistent across all studies however is the inclusion of at least one biomarker from each of the categories of cardiovascular, metabolic, inflammatory, and immune biomarkers. On the other hand, quite a number of factors have been identified to predict high AL scores in different populations, most prominently including race, income, and BMI (7, 23). In this study income was not found to be significantly associated with AL, which is contrary to findings in other studies (27, 31, 32). This may be attributed to the fact that we looked at a subpopulation of women who are in the later stages of their working life such that those who earn more maybe as likely as those who earn less to have health problems that would drive their AL profiles up. We also found a similar relationship between race and AL as established by previous studies, with higher scores reported in Black and Hispanic populations (7).

The strong association that we found between BMI and AL in this study is consistent with most studies (33). Recent meta-analysis studies have offered strong evidence for an association between migraine and obesity likely mediated by gender and migraine frequency (34, 35). These studies have shown an increased risk of having migraine in obese women compared with normal weight women. In addition, obesity is thought to be a risk factor for higher frequency of migraine attacks (36) as well as chronification of migraine (34, 37). Since reduction in weight is thought to be beneficial in reducing the burden of migraine attacks (38), and given the strong association between BMI and AL, it is likely that reduction of BMI may lead to even more significant reduction of allostatic load in perimenopausal migraineurs, something that remains to be confirmed by future studies.

Sleep problems are commonly reported in association with migraine disorders (39), with higher frequency of migraine attacks (40) and chronic migraine (41) being associated with poor sleep quality. Shared nervous system pathways involved in sleep cycle dysregulation and migraine pathophysiology may underlie the association between migraine and sleep disorders (39). There is an increased incidence of sleep disorders among women during perimenopause. The prominence of sleep problems seems to be age-dependent with significantly larger numbers in women older than 50 years (42, 43). In our study we distinguished between three types of sleep problems as there is evidence for differences in their prevalence and their course during the perimenopause transition (44). For instance while waking up several times during the night is the most prevalent sleep problem in women, trouble falling sleep also increases significantly during the perimenopause transition (44). In our study, while we found that the migraineurs experienced more sleep problems than the controls in the univariate analysis, parameter estimates were not significant for the association between each of the three groups of sleep disorders and migraine in our final regression model (Table 3). Since the majority of women in both cohorts were early perimenopausal, we cannot be sure that similar patterns will be observed in the later stages of menopause. Examination of the relationship between migraine and sleep problems during the course of menopausal transition is not within the scope of current study and remains to be examined.

The management of migraine can be very challenging for both clinicians and patients specially in the lack of specific treatment recommendation for perimenopausal women with migraine (6). Several classes of medication are used in the treatment of migraine i.e., anticonvulsants, antihypertensives, antidepressants just to mention a few, throwing more light on the fact that no one drug works best all the time. These medications have varying levels of effectiveness in different patients, which is also an indication that there is substantial influence from patient specific factors (45). Tying this to the findings from this study of perimenopausal women, a holistic look at the management of these patients would include, knowledge that certain races are more prone to the wearing effects of menopause, as well as the fact that obesity significantly impairs homeostasis in these women. Early interventions and targeted attention to addressing problems in this population will go a long way to reduce the burden and sufferings of menopause.

### Limitations

This study is not without its limitations. One of the limitations of our study is that it is a secondary analysis of the data from SWAN study and not designed with the aim of studying migraine disease. Therefore, there are not additional details on the disease clinical characteristics or its associated symptoms. Another limitation of our study is that it relies on self-reported migraine and not a clinical diagnosis meeting the *International Classification of Headache Disorders* (ICHD) (46) criteria. Despite this limitation, self-reports of migraines are still highly specific and modestly sensitive (47) meaning that there are low false positive in self-reported migraines. Any potential underestimation of the migraineurs due to low sensitivity of self-reported migraines may have lowered the significance of the observed differences between the two cohorts. Another limitation of the data used in this study is that there are no specific questions on other headache types in the SWAN data, so if the participants were experiencing non-migraine headaches they may still be included in the control cohort. On the other hand, By looking at just perimenopausal women we limit generalizability of our findings to the larger population. However, our findings are consistent with most studies that looked at allostatic profiles in the general population (29, 48). Again, by giving equal scores to each biomarker we assumed each biomarker contributes equally to determine the stressors on our bodies. Despite these limitations, this study has some strengths such as the fairly good representation of racial groups, relatively large sample size translating into more power, and finally the data to differentiate the specific times during the night women have sleep problems.

## Conclusions

Overall, the results of this study suggests that perimenopausal migraineurs are more likely to have higher allostatic load than their non-migraine counterparts. Given the association between migraine and increase AL score, future studies may consider examining the influence of including an index corresponding to the burden of the migraine disease (e.g., MIDAS score derived from the Migraine Disability Assessment) as a marker for increased allostatic load in the calculation of AL scores. In addition, we need further studies to delineate the neural circuitries associated with sleep disturbances and their association with hormone replacement therapy and allostatic load. Moreover, future studies need to examine how much of the excess risk of high allostatic load is attributed to the various reasons for the disparities; low SES, discrimination, systemic racism, provider bias, individual sensitivity to race just to mention a few, among perimenopausal women with migraine (49).

## Data Availability Statement

The datasets presented in this study can be found in online repositories. The names of the repository/repositories and accession number(s) can be found at: Data for this study was obtained from the Study of Women's Health Across the Nation (SWAN). https://www.nia.nih.gov/research/dgcg/study-womens-health-across-nation-swan-repository.

## Ethics Statement

The Institutional Review Board at the Massachusetts General Hospital approved the secondary data analysis. The Institutional Review Board at each of the SWAN study sites had approved the study protocol, and all participants had provided written informed consent prior to participation in the study.

## Author Contributions

All authors listed have made a substantial, direct and intellectual contribution to the work, and approved it for publication.

## Conflict of Interest

The authors declare that the research was conducted in the absence of any commercial or financial relationships that could be construed as a potential conflict of interest.

## Appendix

**Appendix 1:** Linear Regression models with AL on Migraine and other predictors—Allostatic Load as a continuous variable.

		**Model 1**			**Model 2**		
**Predictor**	**Reference**	**β**	**SE**	***p*-value**	**β**	**SE**	***p*-value**
**Migraine**	Control	0.24	0.11	0.022	0.22	0.72	0.005
**Age**		0.07	0.01	<.001	0.07	0.01	<0.001
**Race**	Caucasian						
Black		0.47	0.10	<0.001	0.46	0.10	<0.001
Hispanic		0.74	0.19	<0.001	0.73	0.19	<0.001
Asian		0.57	0.11	<0.001	0.58	0.11	<0.001
**BMI**	Normal						
Overweight		0.92	0.10	<0.001	0.91	0.10	<0.001
Obese		2.48	0.10	<0.001	2.45	0.10	<0.001
Underweight		−0.21	0.32	0.53	−0.24	0.33	0.455
**Income**	<$19,999						
$20,000–$49,999		−0.21	0.14	0.147	−0.18	0.14	0.205
$50,000–$99,999		−0.31	0.14	0.031	−0.27	0.15	0.060
$100,000 or more		−0.45	0.16	0.006	−0.40	0.16	0.014
**Menopausal status**	Premenopausal						
Early perimenopause		0.11	0.09	0.246	0.09	0.09	0.335
Late perimenopause		0.34	0.21	0.103	0.32	0.21	0.118
Postmenopausal		0.62	0.34	0.066	0.59	0.34	0.082
**Trouble falling asleep**					0.03	0.02	0.054
**Waking up during sleep**					0.01	0.01	0.659
**Waking up earlier**					0.01	0.016	0.628

*R^2^ for model 1: 0.323*.

*R^2^ for model 2: 0.326*.

*β, Standardized regression model; SE, standard error*.
